# Supplementary material for: Predicting Disease in Transition Dairy Cattle Based on Behaviors Measured Before Calving
Source: Animals (Basel). 2020 May 27;10(6):928. doi: 10.3390/ani10060928 (PMC7341500; doi:10.3390/ani10060928)
Supplement: Supplementary file 1 [file animals-10-00928-s001.pdf]

animals

Supplementary: Predicting Disease in Transition Dairy Cattle Based on Behaviors Measured Before Calving

Mohammad W. Sahar, Annabelle Beaver, Marina A. G. von Keyserlingk and Daniel M. Weary

Table S1. Results from the multivariable model assessing water intake data for multi- and primiparous cows combined. Estimates of slope, SE, *P*-value, odds ratio (OR) and 95% confidence interval (CI) are reported. Odds ratios for actor behavior at water bins, and liters of water intake are calculated based on units of 6 actor behaviors, and 1 liter of water intake.

Variable Name	Slope	SE	*P*-value	OR (95% CI)
Multi- and primiparous cows				
Actor behavior at water bins	−0.334	0.161	0.03	0.72 (0.52–0.98)
Liters of water intake	−0.023	0.009	<0.01	0.98 (0.96–0.99)

Table S2. Results pertaining to the predictive ability of the model reported in Table S1 are shown. Sensitivity, Specificity, PPV[1], NPV[2], area under the ROC[3] curve, and overall accuracy were calculated for both, the training[4] and the testing[5] datasets.

Dataset	Sensitivity	Specificity	PPV	NPV	ROC	Overall Accuracy
Training dataset for multi- and primiparous cows	68.04	46.74	68.04	46.74	64.2	57.67
Testing dataset for multi- and primiparous cows	42.86	62.16	42.86	62.16	54.7	52.78

[1] Positive predictive value; [2] Negative predictive value; [3] Receiver operating characteristic; [4] The dataset used for training the model; [5] The dataset used for testing the model.
